# Self-Efficacy, Social Support, and Depression: Mediators of Medication Adherence in Dialysis Patients

**DOI:** 10.3390/healthcare13040425

**Published:** 2025-02-16

**Authors:** Reynita Saguban, Sumathi Robert Shanmugam, Evalynn Rondilla, Joyce Buta, Nuha Ayad H. Alatawi, Richard Maestrado, Sameer A. Alkubati, Romeo Mostoles, Nojoud Abdullah Alrashidi, Maha Sanat Alreshidi

**Affiliations:** 1 College of Nursing, University of Hail, Hail 55473, Saudi Arabia; e.rondilla@uoh.edu.sa (E.R.); j.buta@uoh.edu.sa (J.B.); r.maestrado@uoh.edu.sa (R.M.); alkubatisa@yahoo.com (S.A.A.); n.alrashidi@uoh.edu.sa (N.A.A.); ma.alrashidi@uoh.edu.sa (M.S.A.); 2 Department of Maternity and Pediatric Nursing, College of Nursing, Princess Nourah bint Abdulrahman University, Riyadh 11671, Saudi Arabia; srshanmugam@pnu.edu.sa; 3 Medical Surgical Nursing Department, Faculty of Nursing, University of Tabuk, Tabuk 47512, Saudi Arabia; n.alatawi@ut.edu.sa

**Keywords:** depression, dialysis, health care provider, health outcomes, medication adherence

## Abstract

**Introduction**: Healthcare providers’ understanding of how self-efficacy and social support affect medication adherence and depression in dialysis patients can lead to holistic interventions and improve outcomes. This study aimed to investigate how self-efficacy and social support indirectly influence the relationship between medication adherence and depressive symptoms in patients with chronic kidney disease (CKD) undergoing dialysis. **Methods**: We employed a cross-sectional observational study design with 668 CKD patients from outpatient departments (OPDs) and dialysis centers in the Hail region of Saudi Arabia. The data were collected between April and May 2024. **Results**: The participants had a relatively high level of self-efficacy (median = 82.00/100) and greater perception of social support (median = 75.500/84) with minimal to mild depressive symptoms (median = 15.00/63); however, 50% of participants scored ≥ 5 (out of 10) on the level of adherence to their medication regimen. Depression was prevalent, with a mean score of 5.03 on the PHQ-9 scale, and was positively correlated with nonadherence. Social support and self-efficacy were negatively correlated with depression, and both partially mediated the link between depression and non-adherence. **Conclusions:** This study found that, despite high social support and self-efficacy, a significant number of patients with CKD on dialysis exhibited medication non-adherence. Depression has emerged as a key factor influencing adherence, even in the presence of social support and self-efficacy. These findings suggest that depression is crucial for CKD management. Healthcare providers, owing to their frequent interactions with patients with CKD, are ideally placed to screen for depression and incorporate management strategies into patient care plans. By addressing both the biological and psychological aspects of CKD, they can empower patients to take a more active role in their treatment, ultimately leading to improved health outcomes.

## 1. Introduction

Chronic Kidney Disease (CKD) has become a major problem worldwide, and medication adherence is an important indicator of the successful management of such conditions [1]. Medication adherence has been a problem among CKD patients, with various estimates of non-adherence ranging from 17% to 74% [2]. Failure to adhere in this way can have adverse health consequences at the individual level, be it needless expenditure on healthcare services and high mortality risks [3]. Given that psychological issues are crucial to understanding the reasons for non-adherence to medications, this study assists in understanding how self-efficacy beliefs, social support networks, and depression status mediate adherence to medicines among individuals with CKD. Self-efficacy is the perception or belief that one has regarding one’s capability to execute the actions required to attain a desired goal in health-related behaviors (including medication adherence) [4]. Family, friends, and healthcare providers provide emotional, instrumental, and informational social support, which is critical in managing chronic illness and adherence to medications. Depression is common in patients with CKD, which, while being a problem itself, also affects other areas of health, such as cognitive abilities, motivation, and general health, which in turn will affect medication adherence. Considering the interplay between these factors and drug treatment, this study sought to expand the existing knowledge about the factors that determine the success of treatment in patients undergoing dialysis.

Treatment adherence is an essential parameter of self-efficacy. Bandura defined self-efficacy as a person’s belief in their ability to act in a designated manner. Earlier studies have confirmed that interdependence exists between self-efficacy and medication adherence among patients with chronic hypertension and congestive heart failure [5]. Medication adherence is emphasized when the issues being targeted are degenerative, such as in the case of CKD. Other investigators have postulated that self-efficacy is an important factor that enhances adherence to pharmacotherapy in various populations [6,7]. Depression symptoms and self-efficacy are factors that influence adherence to medication [5]. The indicators of depression are depressive symptoms that are mediated by patients’ self-efficacy toward drug adherence in people with chronic diseases [5]. In other words, when depressed patients are constantly pessimistic and hopeless, they become less prone to sticking to their drug regimen. Conversely, social support increases the probability of adherence to medication among patients with chronic illness [7,8]. Therefore, if clients have enough social networks, it can help them obtain more information about their health to manage diseases better and increase their level of medicine intake [5]. Indeed, social support, as a mediator, narratively explains how depression may impact drug adherence [5].

Support from family members and healthcare workers assists individuals with CKD in complying with therapy [9]. However, no studies have assessed the mediating effects of self-efficacy and social support on medication compliance and depressive symptoms among CKD patients on dialysis. A reverse correlation has been noted between depression and medication compliance among elderly patients with coronary artery disease. It was also established that self-efficacy is a significant mediator in the relationship between depression and medication adherence, in that depression would mean low self-adherence for elderly people with coronary heart disease (CHD). Elderly CHD patients show poor adherence to prescribed treatments, which can lead to adverse complications [5]. In research conducted by Son and Won [10], it was indicated that depression and medication compliance were related within social networks as intervening variables, reasoning that social networks aid in receiving external support from family and friends, which facilitate communication about health coping strategies and promote treatment compliance. Maeda et al. [11] established that self-efficacy had a mediating effect on the relationships between depression, treatment adherence, and social support among heart failure patients. Self-efficacy may then be conceptualized as an intervening variable that can be improved by different types of interventions, such as self-directed care, which aims to improve practice behavior for disease management. Likewise, the relationship between medication adherence and depression has also been mediated in turn by social support and self-efficacy among older depressed patients for whom there is a strong expectation that they would not take the medication once issued to them [5]. Although there has been research on these factors in CKD patients, understanding of the direct effect of self-efficacy and social support as mediators of adherence to medication and elderly patients with depression on dialysis is still limited.

Health professionals are essential for promoting self-efficacy and social support in individuals with CKD. Health professionals can design such interventions because they know how these factors may influence medication adherence and depression. This understanding would first inform strategies designed to increase the levels of self-efficacy and social support among these populations so that depression, a frequently occurring complication, can be reduced. Furthermore, suppose that the research determines how self-efficacy enhances the use of prescribed medications, while networks influence adherence behavior. This enables relevant healthcare service providers to improve the management of CKD patients. This study aimed to investigate how self-efficacy and social support influence medication adherence in CKD patients who may have depressive symptoms. This will help healthcare providers develop specific interventions that can improve adherence to medications by these patients, manage depressive disorders, and enhance treatment outcomes.

## 2. Methods

### 2.1. Study Design

We employed a cross-sectional observational study design that allowed the collection of data on all the studied variables from participants at a single point.

### 2.2. Participants/Setting

The Raosoft online calculator was used to identify the sample size (https://www.calculator.net/sample-size-calculator.html?type=1&cl=95&ci=5&pp=50&ps=5000&x=Calculate, accessed on 23 April 2024), which suggested a minimum sample size of 357. However, we opted to enroll 668 participants using convenience sampling to increase generalizability and account for potential attrition. Included in this study were as follows: (a) 18 years old and older, (b) CKD on dialysis, and (c) willingness to participate. Patients with cognitive impairment who could not provide informed consent were excluded. This study was conducted in the outpatient departments of the four most sought government hospitals in the Hail region of Saudi Arabia.

### 2.3. Data Collection

The researchers collaborated with outpatient department authorities to identify and approach potentially eligible participants. Eligible participants were then provided with a detailed information sheet outlining the goals, procedures, potential risks, and benefits of the study. After the participants carefully reviewed this information, they were given time to decide whether to participate. The participants who chose to participate provided informed consent before answering the paper-based questionnaire. The data were collected between April and May 2024.

### 2.4. Questionnaire

In this study, four questionnaires were adapted. These include the following:

Efficacy in managing CKD was determined with a scale that measured self-efficacy in patients using the Chronic Kidney Disease Self-efficacy (CKD-SE) scale [12]. The scale has 25 items that are further divided into four subdivisions: autonomy, self-integration, problem solving, and seeking social support.

The Multidimensional Scale of Perceived Social Support [13] is a self-report scale comprising 12 items that seek to gather data on social support. This type of scale is self-administered, and respondents rate their responses to questions using a Likert scale ranging from “strongly disagree” to “strongly agree”. The scoring procedure involved calculating subscales for family, friends, and significant others by totaling item scores belonging to the respective groups and then dividing this number by the number of items in each subscale. All 12 items were summed to obtain a total score. In general, a greater score on a subscale or total score corresponds to a greater perceived level of social support from that particular subgroup or for the total group, respectively.

The 10-item Medication Adherence Rating Scale (MARS) [14] is a self-assessment tool intended to evaluate the level of medication adherence in patients. Instead of using a traditional Likert scale with different degrees of agreement, it used a simple yes/no type. Each question relates to medication-taking behavior. Scoring was 1 point for each “yes” answer and 0 for each “no” response. The final score is computed by summing all the points from all the questions which is ten in number. Higher scores closer to 10 indicated poorer adherence to medication regimens, whereas lower scores indicated better adherence.

The Beck Depression Inventory—Second Edition (BDI-II) is a widely used tool for measuring the severity of depression symptoms experienced in the past two weeks [1996]. The 21-question self-administered questionnaire asked individuals to choose the statement that best reflected their feelings. Each question had four answer choices representing increasing levels of depression severity, and these choices were assigned point values (typically from 0 to 3). The total score was calculated by summing the points from all the questions. While higher scores indicate a greater likelihood of depression, a formal diagnosis should always be made by a qualified mental health professional and should not rely solely on BDI-II scores [15].

While these four tools have been extensively used in research, their appropriateness for use in the Saudi Arabian context has been established. To achieve cultural equivalence, we translated the tools forward and backward using a language expert. This process minimizes misunderstandings caused by cultural nuances or idiomatic expressions. The involvement of four experts in the field to validate the adapted questionnaires bolsters this study (two are psychometricians in the field, one is a research professor, and one is a research assistant in the hospital). These experts examined the content of the questions and ensured that the items reflected the targeted constructs among populations living in Saudi Arabia. Testing of reliability was done using 20 participants for evaluating internal consistency, resulting in Chronbac’s alpha of 0.79 for the CKD-SE, 0.81 for the Multidimensional Scale of Perceived Social Support, 0.77 for the Medication Adherence Rating Scale (MARS), and 0.83 for the Beck Depression Inventory—Second Edition (BDI-II).

#### Ethical Consideration

This study was approved by the Institutional Review Board of the University of Hail (IRB-H-2023-10) to ensure that the study was ethical. The participants’ privacy, anonymity, and confidentiality were ensured. Informed consent was obtained from all participants, and they were free to discontinue their participation in the study whenever they wished, without facing any consequences.

### 2.5. Data Analysis

SPSS version 26 (IBM, Armonk, NY, USA) was used for data analysis. Participant characteristics concerning medication adherence, depressive symptoms, self-efficacy, and social support were descriptively analyzed. Categorical variables are expressed in terms of frequencies and percentages. Further, path analysis or structural equation modeling (SEM) was employed to analyze the hypothesized self-efficacy and social support strains as mediators of the effect of medication adherence on depressive symptoms. This analysis examined the direct relationship between medication adherence and depressive symptoms and, therefore, disregarded self-efficacy and social support. Self-efficacy and social support rationale—the proportion of depressive symptom variance accounted for by medication adherence by self-efficacy and social support. The statistical significance for all tests was set at *p* < 0.05.

## 3. Results

Table 1 presents the participants’ characteristics. The majority of participants were aged >35 years (59.7%), with a mean age of 35.61 ± 9.67 years (Range: 22.0–59.0 years), and males (68.7%). More than half were married (54.2%), and the majority held a high school degree (42.2%), followed by a college degree (40.3%). Most respondents (62.6%) had 10,000 Saudi Arabian Riyals (SAR) income or less. This family income likely represents a middle-income range for families in the Hail region based on general observations and considering the cost of living in the area.

The participants had a relatively high level of self-efficacy (median = 82.00/100) and greater perception of social support (median = 75.500/84) with minimal to mild depressive symptoms (median = 15.00/63); however, 50% of participants scored ≥5 (out of 10) on the level of adherence to their medication regimen (Table 2).

Self-efficacy was significantly and positively related to social support of the participants (r = 0.327, *p* < 0.001). A notable correlation was found between self-efficacy and medication adherence (r = 0.121, *p* = 0.002). Additionally, in the reverse direction, negative correlations between depression and medication adherence with the social support of participants were obtained (r = −0.260, *p* < 0.001, and r = −0.091, *p* = 0.018, respectively). Finally, self-efficacy was negatively correlated with depression (r = −0.083, *p* = 0.033). It is worth noting that depression was negatively correlated with medication adherence (r = −0.408, *p* < 0.001) (Table 3).

Multiple linear regression analysis of the variables as predictors of medication adherence among patients with CKD undergoing dialysis showed that the model was significant (*p* < 0.001). This accounted for 27.5% (R^2^ = 0.275, adjusted R^2^ = 0.263) of the variance in medication adherence. Compared to the reference categories, being age 26–35 or more than 35 (*p* < 0.001), having income between 10,001 and 20,000 (*p* < 0.001), self-efficacy (*p* < 0.001), and depression (*p* < 0.001) were significant predictors of medication adherence among patients with CKD undergoing dialysis (Table 4).

## 4. Structural Equation Modeling

**Figure 1:**
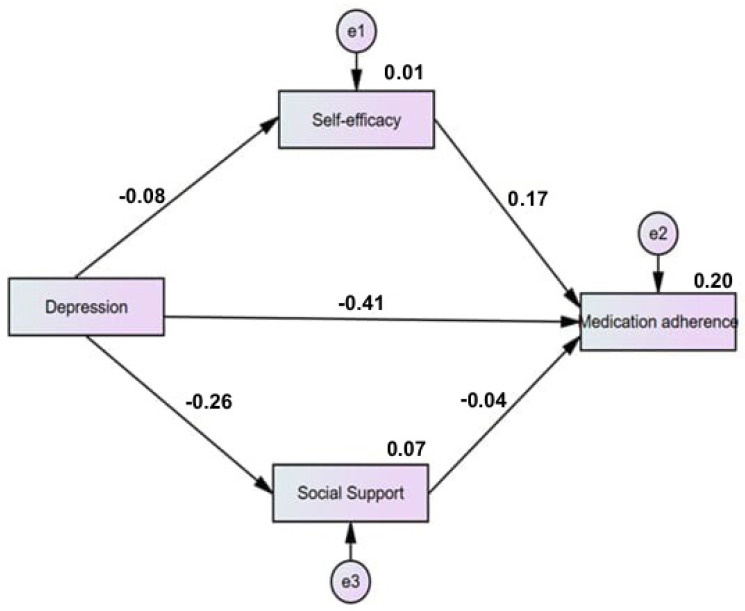
Mediating effect of self-efficacy and social support on the relationship between depression and medication adherence. The path model demonstrates that depression has a detrimental impact on medication adherence, both directly and indirectly through its influence on self-efficacy. Direct effects reveal that higher levels of depression are linked to lower levels of medication adherence (−0.41) and social support (−0.26). Conversely, higher levels of self-efficacy are associated with increased medication adherence (0.17). Indirectly, depression negatively affects medication adherence by impacting self-efficacy (−0.08) (Legend: Numbers on arrows represent standardized coefficients (path coefficients); e1, e2, and e3 represent error terms).

Table 5 illustrates the direct, indirect, and total effects of depression on medication adherence mediated by social support and self-efficacy among patients with CKD undergoing dialysis. The total effects indicated that depression significantly influenced medication adherence (β = 0.408, *p* < 0.001) (Figure 1).

## 5. Discussion

This study aimed to investigate how self-efficacy and social support indirectly influence the relationship between medication adherence and depressive symptoms in CKD patients undergoing dialysis. This study found that the respondents had a reasonably high median level of self-efficacy, perception of social support, and depressive symptoms that were minimal to mild. Furthermore, some patients in this study may not have taken their medications as prescribed. This observation implies complex interrelationships between self-efficacy, social support, depressive symptoms, and medication compliance among the respondents. In addition, the findings of a recent study on CKD patients able to use medication were in line with the previous findings relating to the medication adherence of elderly patients with CKD and the relationship between psychosocial and medication aspects. In comparison with this research, Nagar et al. [16] and Sharma and Khatiwada [17] continue to see a high percentage of reported depression among patients with CKD, which corroborates other areas where people with this type of illness tend to suffer the same major psychological difficulties. Another study on the relationships between self-efficacy, social support, and adherence behaviors in patients with CKD [18] pointed out how self-efficacy and resilience of family members can play a pivotal role in ensuring patient compliance. Moreover, the study by Lee et al. [19] examined strategies for interventions and therapy aimed at addressing factors that decrease quality of life, cause changes in self-care behavior, and cause depressive symptoms in CKD patients on hemodialysis, proposing integrated care solutions. The management of CKD involves biological and emotional factors that need to be addressed using an integrated approach. Healthcare providers may assist in targeting interventions that enhance patient performance and augment treatment outcomes. This may include providing educational resources to enhance self-efficacy, promoting interaction to strengthen social relations, and addressing issues related to compliance with drug therapy.

The present investigation revealed that social support, self-efficacy, depression, and medication use are powerful predictors of medication adherence. More specifically, among the three associations, the relationship between social support and self-efficacy as well as between self-efficacy and medication adherence are major determinants of adherence behaviors. The findings of the present study are also consistent with those of several other authors who recognized social support systems, depression, and self-efficacy as having important roles in determining drug usage patterns. Asadizaker et al. [20] also observed that more support tending to less depression led to more self-efficacy and better health perception, which in turn led to better compliance behavior in hemodialysis patients, thus stressing the importance of these variables in compliance behavior within other health problems. These results coincide with those of Paterson et al. [21], who reported on medication compliance among patients who had renal transplants, suggesting a direct correlation between neurocognitive abilities as typically measured, such as self-efficacy, and depressive symptoms. This emphasizes the role of cognitive and psychosocial factors in predicting adherence behaviors and emphasizes self-efficacy, depression, and social support as factors resulting in medication nonadherence. As a further example, Cha et al. [22] studied the mediating role of depressive symptoms in the relationship between self-efficacy for medication use and self-reported adherence to antiretroviral therapy (ART) in HIV-infected patients. The study found intricate relationships between social support systems, self-efficacy, depressive syndrome, and compliance with medication and argued that all such factors should be incorporated at the same time when strategies for better compliance are devised.

The present research established that social support, self-efficacy, depression, and medication adherence are intertwined issues. It is worth noting that social support, self-efficacy, and adherence to medication have positive relationships and explain some of the adherence behavioral dimensions. Moreover, these results are in line with the findings of other authors who claimed for the given case the importance of social support, depression, and self-efficacy in drug-taking behaviors. According to a study by Asadizaker et al. [20], the interaction effects of social support, depression, perceived self-efficacy, and perceived health are significantly influential in enhancing treatment compliance among hemodialysis patients. This underlines the importance of these variables in adherence behaviors in a number of diseases, which is consistent with the findings of Paterson et al. [21], who studied compliance with medication in patients who had a renal transplant. Their results most precisely gauged the mean self-efficacy or depression of individuals using traditional measures. This highlights the significance of cognition and psychosocial attributes in predicting adherence behaviors and views self-efficacy, depression, and social support as fundamental reasons for noncompliance to taking medication. Moreover, Cha et al. [22] investigated how depressive symptoms acted as a mediator between self-efficacy in medication use and self-reports of ART adherence among infected HIV patients. Therefore, social support systems, self-efficacy, depressive syndrome, and medication adherence should be considered simultaneously when planning and implementing measures to improve compliance. To reduce the external stresses around poverty, which can result in poor self-efficacy and low motivation in a CKD patient, providers must push for the implementation of policies that expand resources such as access to medical transport or free prescriptions. When focusing on improving self-efficacy and social support in CKD patients while trying to tackle their depression, one needs to first understand that it is a multifactorial disease and hence requires team-based intervention, including educational programs, peer support groups, mental health assessment and treatment, and addressing social issues. Such solutions can help patients to increase their compliance with therapy and their lives in general.

Depression had a negative impact on both social support and self-efficacy. This suggests that depressed patients expressed low social support due to their friends and family, as well as decreased trust in their ability to cope with health issues. Surprisingly, depression had a direct and positive impact on prescribed medications. Such a conclusion can be based on two theories: sicker patients make more visits to the doctor, or they place more importance on health matters. In contrast to these findings, Cukor et al. [23] noted that the prevalence of depression is a serious adherence factor in patients with CKD, proving that adherence to treatment is subject to psychological factors. Lee et al. [24] investigated the impact of self-management programs on medication adherence among elderly patients and suggested that the use of multifaceted approaches may foster adherence ambitions in patients with long-standing illnesses. In line with this, Murali et al. [25] examined the reasons for low adherence to medication among patients with CKD, which included but were not limited to low health literacy, depression, and pill burden, thus offering explanations for various medication adherence challenges. These studies highlight the complex interrelation of depression, social support, self-efficacy, and adherence to medications among CKD patients, indicating that there is a need for well-defined interventional strategies that encompass both the psychological and the clinical aspects of care at the same time. Depression and low self-esteem are essential factors to consider if providers wish to enhance medication adherence and general health among their CKD clients; thus, appropriate screening for both of them should be conducted. Conclusively, integrating such evaluations as part of CKD management enables timely treatment that focuses on both the patient’s mental state and the management of the chronic illness. It is clear that the interventions can enhance self-efficacy and self-management of social support in patients with CKD. All such measures are leaning not only to combat depressive symptoms but, more importantly, to encourage people to take an active part in health care by improving their adherence to medication and general results.

The findings of this study reveal that social support and self-efficacy work independently to enhance compliance with medication use. Medication compliance was associated with individuals in stronger social support groups and those with greater self-efficacy in controlling their diseases. This result highlights the importance of psychological and social determinants of adherence behaviors among persons with CKD. In relation to other works, ref. [18] emphasized the correlation between self-efficacy, social support, and family resilience among CKD patients on hemodialysis and thus highlighted the beneficial effect of these factors on adherence. In addition, Mousa et al. [26] studied other variables, such as dialysis-related factors that interfere with self-efficacy and quality of life in patients on hemodialysis, indicating that self-efficacy has different dimensions in the management of CKD. On the other hand, Chuang et al. [27] focused on the effect of knowledge and self-management in patients with early-stage CKD, stressing mental health as a mediating variable between self-management behavior and life enjoyment. Collectively, there is a general consensus among researchers that there are interactions between medication-taking behavior and gaps that constantly help out a person’s medication-taking ability. To this end, social support networks and self-efficacy while dealing with depression among patients with CKD can be improved by specific measures by healthcare providers. Health coaching, education programs, peer support, and standard mental health assessments can help in formulating comprehensive care that incorporates psychological and social factors affecting compliance. Such interventions not only assist patients but also create a culture of support that enhances the health of patients.

Findings suggest that higher levels of depression are linked to lower levels of medication adherence and social support. Conversely, higher levels of self-efficacy are associated with increased medication adherence. Indirectly, depression negatively affects medication adherence by impacting self-efficacy. To some extent, depression increases the challenges one faces in medication adherence for individuals who possess low self-efficacy. Research has shown that depression, regardless of its severities and forms, is associated with a greater risk of developing poor medication adherence in almost all chronic diseases. For example, studies have indicated that depressed individuals do not adhere to their medications for diabetes [28], HIV/AIDS [29], or even heart failure [10,30]. In particular, it was emphasized that with regard to negatively mediating depression and medication adherence in type 2 diabetic patients, some social factors can lessen the impact, thus social factors are helpful though alone not sufficient [29]. Likewise, an earlier study further illustrated how the intricacy of medication schedules may worsen adherence problems in depressed patients, suggesting that more targeted approaches are required to solve some of the issues indicated [30]. Self-efficacy also stands out in this context: the higher self-efficacy individuals have, the greater their medication adherence [10]. This means that depressed patients may benefit from self-efficacy-enhancing interventions aimed at improving adherence. For instance, self-efficacy was found to mediate the relationship between depression and medication adherence from the treatment compliance perspective which highlights the role of psychology in treatment compliance [10]. Such claims are consistent with results obtained when they considered the influence of psychosocial factors as well as self-efficacy on medication adherence after acute coronary syndrome [31]. Furthermore, these results have greater implications than those of individual patient care. The complex nature of depression’s effect on medication noncompliance speaks for integrated care models that target mental health and chronic disease treatment. For example, researchers argued that maintenance treatment of depression is necessary for better compliance with anti-hypertensive medication and recommended the use of psychosocial and medical therapy [32]. This is of paramount importance, especially to groups that are known to be highly noncompliant, such as the elderly or those with multiple chronic illnesses [33,34].

These results suggest that social support and self-efficacy may have mediating effects; however, depression had a significant effect on drug compliance. Hence, other variables may explain the relationship between depression and compliance with drug therapy, which warrants further research. They will also analyze how literacy levels affect the health, depression, and pill burden of patients suffering from kidney disease, which makes them noncompliant with medications. The final aspect worth evaluating involves the effectiveness and quality of life measures of CKD patients and was detailed in the work of Fradelos et al. [35], who assessed the effectiveness of spirituality on the three determinants of mental health: stress, anxiety, and depression in women suffering from renal diseases. Elkheir et al. [36] regarded this study as a “point in time” examination of depression symptoms and determinants among renal patients on dialysis, and the results revealed that there was a higher prevalence of depressive disorders among subjects on dialysis. The results of the present study and those of earlier studies may provide more information about the relationship between depression and social support, self-efficacy, and adherence to medication among patients with CKD. It would be more reasonable to combine these factors in such a way that the end result of treatment would be satisfactory while enhancing the life conditions of patients with CKD. Considering the complex consequences of depression while also understanding the role of support and self-efficacy factors would enable professionals to develop interventions that cover all these areas. This might include embedding mental health screenings, linking clients with social services and relevant support groups, providing medication compliance education, and distributing materials appropriate to people’s literacy skills.

## 6. Study Limitations

There are several limitations and factors that would undermine the generalizability of the findings of this study. First, the cross-sectional nature of the study design is its limitation in providing the ability to determine the direction of causality among the variables under relativeness. Depression can also affect social support and self-efficacy. Second, we collected the data using convenience sampling, focusing on only a few hospitals in the Hail region, which raises an important issue regarding the representativeness of the subjects to all CKD dialysis patients. This shortcoming impedes the extension of the findings of this study to other countries and populations. Third, the self-report measures in our study of medication adherence, depressive symptoms, self-efficacy, and social support increased exposure to recall and social response biases. Participants are likely to have forgetfulness toward some details of their adherence or simply give the “correct” answer. While it is also true that the deployment of already-made questionnaires can be an advantage, it is imperative to analyze the context in which they are being used and the extent to which they are appropriate. Moreover, the unexpected finding of higher medication adherence in higher-income groups may be confounded by factors such as access to specialized healthcare and health literacy, which may vary across income levels. Although further stratification of the elderly population into more specific age groups is desirable, we opted for broader age categories to ensure adequate sample sizes within each group, which is crucial for robust statistical analysis. Finally, our analysis failed to control for all possible interceding factors that may adjust for the association between depression and medication adherence. The lack of these controls could have resulted in such questions being included in the results. To address these limitations, future research should consider longitudinal designs to assess the causal effects of the intervention, representative sampling techniques to enhance generalization, and the inclusion of objective indicators of medication adherence, levels of depression and self-efficacy, and social support. In addition, researchers ought to control a greater number of essential elements in their undertaking, thus improving the reliability of their discoveries.

## 7. Study Strengths and Application

This study advances the understanding of CKD management by exploring the complex interactions between depression, self-efficacy, and social support, which collectively affect treatment adherence. The findings also support the argument for an inclusive model that addresses cognitive, sociopsychological, and clinical aspects, thus providing wide margins for coming up with appropriate responses. One of the most important advantages of this study is the ability of the authors to ground procedures and reassure emphasis on self-efficacy and social support in the context of chronic condition management, as well as the separation of depression and medication adherence through these factors. Taking the form of routine monitoring of the basic mental status of patients suffering from CKD, the inclusion of focused training courses aimed at improving health literacy and skill of self-management, involvement in the support network, incorporating self-management into routine care, and improving patient involvement through technology. Overall, these strategies empower patients, change their behavior with respect to adherence, and eventually improve health outcomes with respect to CKD management.

## 8. Conclusions

While this study highlights depression as a major barrier to CKD patients’ adherence to prescribed medication in Hail City, Saudi Arabia, these findings cannot be generalized to all CKD patients. Considering the peculiarities of our sample, such as high self-efficacy and social support, the relationship between depression and non-adherence observed in the study may be different in other settings. Our study emphasizes the need to manage depression as part of the management of CKD. Healthcare providers who work with these patients over a long period can identify and provide treatment for depressed moods. They agree to strike a balance, as they integrate depression interventions into the treatment of their patients for better outcomes.

## Figures and Tables

**Table 1:** Characteristics of the participants. N = 668.

Variable		n	%
Age	25 or less	195	29.2
	26–35	74	11.1
	More than 35	399	59.7
	Mean ± SD: 35.61 ± 9.67		
	Range: 22.0–59.0		
Gender	Male	209	31.3
	Female	459	68.7
Marital status	Single	306	45.8
	Married	362	54.2
Educational level	High school	282	42.2
	Diploma	91	13.6
	College	269	40.3
	Master	26	3.9
Income	10,000 SAR or less	418	62.6
	10,001–20,000 SAR	197	29.5
	More than 20,000 SAR	53	7.9

**Table 2:** Level of self-efficacy, social support, depression, and medication adherence.

Variable	Min.	Max.	Median	Mean	Standard Deviation
Self-efficacy	25.000	100.000	82.000	85.000	10.000
Social support	26.000	84.000	75.500	72.000	15.000
Medication adherence	0.000	10.000	5.000	5.8.000	2.500
Depression	0.000	63.000	15.000	18.000	12.000

**Table 3:** Correlation matrix between the studied variables.

Variables		Self-Efficacy	Social Support	Medication Adherence	Depression
Self-efficacy (out of 100)	Pearson’s r	1			
	*p*-value				
Social support (out of 84)	Pearson’s r	0.327 **	1		
	*p*-value	<0.001			
Medication adherence (out of 10)	Pearson’s r	0.121 *	−0.091 *	1	
	*p*-value	0.002	0.018		
Depression (out of 63)	Pearson’s r	−0.083 *	−0.260 **	−0.408 **	1
	*p*-value	0.033	<0.001	<0.001	

** Correlation is significant at the 0.05 level (two-tailed). * Correlation is significant at the 0.01 (two tailed).

**Table 4:** Multiple linear regression of predictors of medication adherence among patients with chronic kidney disease undergoing dialysis.

Predictors *	β	S.E	t	95% CI for β	*p*-Value
Age					
25 or less	Reference				
26–35	−1.074	0.288	−3.726	−1.998–−0.855	<0.001
More than 35	−1.207	0.202	−5.985	−2.763–−1.668	<0.001
Gender					
Male	Reference				
Female	0.273	0.182	1.831	−0.024–0.678	0.133
Educational level					
High school	Reference				
Diploma	−0.418	0.256	−1.383	−0.841–0.146	0.103
College	−0.134	0.208	−1.446	−0.705–0.107	0.521
Master	0.674	0.461	2.001	0.017–1.802	0.144
Income					
10,000 SAR or less	Reference				
10,001–20,000 SAR	0.627	0.196	3.196	0.242–1.012	<0.001
More than 20,000 SAR	−0.627	0.334	−1.877	−1.284–0.029	0.105
Self-efficacy	0.034	0.007	5.041	0.021–0.047	<0.001
Social support	0.006	0.007	0.812	−0.009–0.021	0.477
Depression	0.053	0.005	11.207	0.044–0.062	<0.001

* R^2^ = 0.275, adjusted R^2^ = 0.263, *p* ˂ 0.001; CI, confidence interval.

**Table 5:** Direct, indirect, and total effects of depression on medication adherence mediated by social support and self-efficacy among chronic kidney disease patients undergoing dialysis.

	Estimate	S.E.	C.R.	*p*
Direct Effects				
Depression → Social support	−0.260	0.025	−6.958	<0.001
Depression → Self-efficacy	−0.083	0.027	−2.144	0.032
Depression → Medication adherence	0.411	0.005	11.387	<0.001
Social support → Medication adherence	−0.039	0.007	−1.092	0.275
Self-efficacy → Medication adherence	0.168	0.007	4.823	<0.001
Indirect Effects				
Depression → Social support → Medication adherence	−0.018	0.008	−2.371	0.018
Depression → Self-efficacy → Medication adherence	0.023	0.007	3.161	0.002
Total Effects				
Depression → Medication adherence (Combined)	0.408	0.005	11.543	<0.001

## Data Availability

The datasets generated in this study are available from the corresponding author upon request.
